# Multicenter Validation of a Risk Classification Cluster for Unfavorable Pathology in Prostatectomy Specimens of Patients with an Active Surveillance Expanded Inclusion Criteria

**DOI:** 10.3390/diagnostics16132073

**Published:** 2026-07-02

**Authors:** Maria Graus Romero, Cristobal Cobo Díaz, Guillermo Lendínez Cano, Laura Chamorro Castillo, Jorge Andres Gutiérrez Suarez, Juan Pablo Campos Hernández, Bernardo Herrera Imbroda, Rafael Angel Medina López, Enrique Gómez Gómez

**Affiliations:** 1 Urology Department, Reina Sofía University Hospital, Maimonides Institute of Biomedical Research of Cordoba (IMIBIC), University of Cordoba (UCO), 14004 Cordoba, Spain; mariagrausromero@gmail.com (M.G.R.); lauracc29@gmail.com (L.C.C.); juanp.campos.sspa@juntadeandalucia.es (J.P.C.H.); 2 Urology Department, Virgen de la Victoria University Hospital, Genitourinary Oncology Translational Research Unit, Institute of Biomedical Research of Malaga (IBIMA Plataforma BIONAND), 29010 Malaga, Spain; crisjecodi07med@gmail.com (C.C.D.); ber.urologia@gmail.com (B.H.I.); 3 Urology Department, Virgen del Rocío University Hospital, Institute of Biomedical Research of Seville (IBIS), University of Seville, 41013 Seville, Spain; glendinez@gmail.com (G.L.C.); rafamedina68@gmail.com (R.A.M.L.); 4 Faculty of Medicine, University of Cordoba (UCO), 14004 Cordoba, Spain; gutierrezsuarezja@gmail.com

**Keywords:** prostate cancer, active surveillance, Gleason score, multiparametric magnetic resonance imaging

## Abstract

**Background:** Active surveillance (AS) criteria in prostate cancer (PCa) are expanding to include selected patients with intermediate-risk features. This multicenter retrospective study aimed to validate a proposed risk group (RG) classification for predicting unfavorable pathology (UP) in radical prostatectomy specimens among patients eligible for AS under expanded criteria. **Methods:** Patients from three Andalusian university hospitals who met the AS criteria, defined as prostate-specific antigen (PSA) ≤ 20 ng/mL, International Society of Urological Pathology (ISUP) ≤ 2, and clinical stage ≤ cT2, were included. The patients were stratified into five RGs according to PSA density, Prostate Imaging Reporting and Data System score (PI-RADS), and clinical stage. UP was defined as ≥pT3a and/or pN+ and/or ISUP grade ≥ 3. **Results:** A total of 244 patients were analyzed. The median age was 63 years, the median PSA 5.98 ng/mL, and the median PSA density was 0.14 ng/cc. UP was identified in 47.1% of radical prostatectomy specimens, increasing progressively across RGs from 20.8% to 93.3%. Each incremental RG was associated with a higher risk of UP, with an odds ratio of 2.14 and moderate predictive accuracy, as reflected by an area under the curve of 0.70. **Conclusions:** The proposed RG classification showed moderate predictive capacity for UP and may improve risk stratification in intermediate-risk patients considered for AS.

## 1. Introduction

Prostate cancer (PCa) is currently the most frequently diagnosed malignancy and the third leading cause of cancer-related mortality among men in Europe [1]. Active surveillance (AS) is the standard management strategy for patients with low-risk (LR) localized PCa and is also recommended for carefully selected patients with intermediate-risk (IR) disease [2]. The primary objective of AS is to delay or avoid the adverse effects associated with definitive local treatment in patients with a life expectancy of at least 10 years who do not require immediate intervention [3]. Patients managed with AS are closely monitored through structured protocols, including prostate-specific antigen (PSA) testing, digital rectal examination (DRE), repeated prostate biopsies, and multiparametric magnetic resonance imaging (mpMRI) [4]. Curative treatment is initiated when predefined clinical, radiological, or pathological criteria indicate disease progression, thereby allowing timely intervention with curative intent [5].

The inclusion criteria for AS programs are designed to identify patients in whom deferring definitive treatment does not compromise oncological outcomes. Owing to favorable long-term oncological results, indications for AS have progressively expanded from very low-risk disease to include selected patients with LR and IR PCa [6]. Robust long-term data from randomized population-based cohorts have demonstrated the oncological safety of AS in screen-detected LR and selected IR disease, with treatment-free survival reaching 43% after 20 years of follow-up [7]. In addition, contemporary real-world evidence from Spanish cohorts has confirmed the feasibility and oncological safety of AS in routine clinical practice, with progression-free survival of 51% at 5 years of follow-up [8].

Several meta-analyses have shown that patients with favorable IR PCa achieve short- and medium-term metastasis-free survival and overall survival rates that are nearly equivalent to those observed in LR patients [9]. In most studies, cancer-specific mortality is reported to be <4%, while 5- to 10-year metastasis-free survival exceeds 95–98% [10]. However, AS in unselected patients with IR PCa has been associated with a higher risk of metastasis compared with patients with low- or very low-risk disease [11]. These higher progression rates may be partly explained by the fact that many evaluated cohorts were managed before the widespread implementation of contemporary risk stratification tools [12]. Therefore, careful selection of patients with favorable IR features remains essential when considering AS.

Current recommendations for AS eligibility are reflected in the DETECTIVE consensus [13], which supports the inclusion of patients with International Society of Urological Pathology (ISUP) grade group ≤ 2 and clinical stage ≤ cT2. The use of mpMRI is also recommended, with lesion characterization based on the Prostate Imaging Reporting and Data System (PI-RADS), which serves as an indirect surrogate for tumor volume. However, the level of evidence supporting these recommendations remains limited, and some patients who do not strictly meet conventional criteria may still have a low risk of disease progression [14].

Prospective data from Asian cohorts have evaluated mid- to long-term outcomes of AS in patients with IR PCa [15]. These data suggest that, when carefully selected, patients with IR disease do not show statistically significant differences in pathological reclassification, disease persistence, or subsequent therapeutic intervention compared with LR patients. Consistently, Baboudjian et al. [16], in a contemporary European cohort, reported favorable oncological outcomes in patients with ISUP grade group 2 PCa selected using mpMRI before image-guided biopsy.

Based on this background, Baboudjian et al. [17] proposed a risk stratification model for patients with IR PCa based on unfavorable pathology (UP) identified in radical prostatectomy (RP) specimens. The present study performs an independent multicenter validation of the risk stratification model and evaluates its association with established prognostic parameters.

## 2. Materials and Methods

### 2.1. Study Design

A multicenter observational and retrospective study was conducted to validate a risk stratification system for UP in prostatectomy specimens in patients with AS expanded inclusion criteria. This study was approved by the local ethics committee (No. 370, Ref. 6123).

### 2.2. Cohort and Procedures

Patients with histologically confirmed PCa who underwent preoperative mpMRI and subsequent radical prostatectomy between February 2017 and January 2024 were included in the study. Inclusion criteria were biopsy ISUP grade group 1–2, prostate-specific antigen (PSA) level < 20 ng/mL, and clinical stage cT1–T2. Patients whose mpMRI was not assessed according to the PI-RADS v2.1 classification or who had missing data for variables required to analyze the primary outcomes were excluded.

All patients underwent preoperative mpMRI and prostate biopsy, including both systematic and image-targeted cores when PI-RADS scores were ≥3, followed by RP with or without pelvic lymph node dissection (PLND) according to individual risk. mpMRI acquisition and prostate biopsies were performed using either a transrectal or transperineal approach, at the discretion of the treating urologist and in accordance with institutional practice.

Histopathological evaluation was performed locally at each participating center by dedicated genitourinary pathologists according to contemporary ISUP recommendations. No centralized pathological review was performed. After prostatectomy, the patients were followed according to institutional clinical practice, with follow-up visits every 6 months for the first 3 years and annually thereafter.

### 2.3. Variables and Outcomes

The primary outcome of the study was the presence of UP in the RP specimen, defined as ≥pT3a and/or pN+ and/or ISUP grade ≥ 3 on histopathological examination. Biochemical recurrence was defined as a postoperative PSA level > 0.2 ng/mL with subsequent confirmatory elevation.

Patients were stratified into five risk categories according to the classification proposed by Baboudjian et al. [17]. The stratification parameters included PI-RADS score, prostate-specific antigen density (PSAD), and DRE findings.

- Risk Group 1 (RG1; very low risk): PI-RADS ≤ 3 and PSAD < 0.15.
- Risk Group 2 (RG2; low risk): PI-RADS 4 with PSAD < 0.15.
- Risk Group 3 (RG3; intermediate risk): PI-RADS 1–4 and PSAD ≥ 0.15.
- Risk Group 4 (RG4; high risk): PI-RADS 5 and normal DRE.
- Risk Group 5 (RG5; very high risk): PI-RADS 5 and abnormal DRE (≥cT2).

### 2.4. Statistical Analysis

Statistical analysis was performed in a stepwise manner. Descriptive statistics are presented as absolute and relative frequencies for categorical variables and as medians with interquartile ranges (IQRs) for continuous variables. Comparisons between groups were performed using the chi-square test or Fisher’s exact test for categorical variables, as appropriate. Continuous variables were compared using Student’s *t* test or the Mann–Whitney U test, depending on data distribution.

Univariable logistic regression analyses were performed to evaluate the association between risk group and unfavorable pathology. First, risk group was modeled as an ordinal predictor to estimate the odds ratio associated with each one-level increase in risk group. Subsequently, a categorical logistic regression analysis was performed using RG1 as the reference category, allowing direct estimation of odds ratios (ORs) and 95% confidence intervals (CIs) for each risk group relative to RG1.

Model discrimination was assessed using receiver operating characteristic (ROC) curve analysis and quantified by the area under the curve (AUC). Model calibration was evaluated by comparing predicted and observed probabilities of unfavorable pathology. Clinical utility was assessed using decision curve analysis (DCA). Recurrence-free survival was estimated using the Kaplan–Meier method and compared between groups using the log-rank test. All statistical analyses were performed using SPSS version 28.0 (IBM Corp., Armonk, NY, USA).

## 3. Results

### 3.1. Cohort Description

The baseline characteristics of the study cohort are summarized in Table 1. A total of 244 patients met the inclusion criteria. The median age was 63 years, and the median PSA level was 5.98 ng/mL (interquartile range [IQR], 4.63–8.69). Most tumors were non-palpable, corresponding to clinical stage cT1c in 83.2% of patients, and were associated with PI-RADS 4 lesions on mpMRI in 50.8% of cases. Most patients had ISUP grade group 2 disease on biopsy (59.8%). UP was identified in 115 patients (47.1%) after RP. The baseline characteristics stratified by participating center are presented in Supplementary Table S1.

### 3.2. Probability of Unfavorable Pathology

When risk group was modeled as an ordinal predictor, each one-level increase in risk group was associated with a 2.14-fold increase in the odds of unfavorable pathology (OR 2.141, 95% CI 1.639–2.795; *p* < 0.001). In the categorical analysis, using RG1 as the reference category, the odds of unfavorable pathology increased progressively across risk groups, with all groups showing significantly higher odds than RG1. The highest-risk group (RG5) exhibited a 47.6-fold increase in the odds of unfavorable pathology (OR 47.600, 95% CI 8.296–273.112; *p* < 0.001). These findings demonstrate a strong stepwise association between increasing risk group and the likelihood of unfavorable pathology (Table 2).

**Figure 1:**
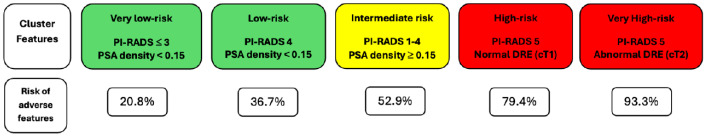
The probability of adverse pathology increases progressively according to RG (risk groups). Chi-square test *p* = 0.000. Abbreviations: PI-RADS—Prostate Imaging Reporting and Data System; PSA—prostate-specific antigen; DRE—digital rectal examination.

Based on the predictive model, the estimated probability of UP ranged from 20.8% in RG1 to 93.3% in RG5 (Figure 1).

### 3.3. Discriminative Capacity of the Proposed Risk Group Model

**Figure 2:**
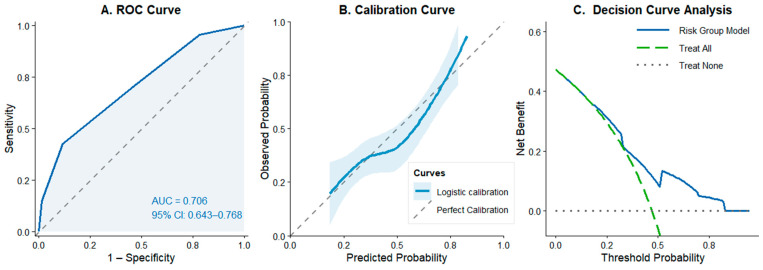
Evaluation of the predictive capacity and clinical utility of the fitted model. (**A**) Discriminatory capacity evaluated by ROC (receiver operating characteristic) curve analysis. (**B**) Model calibration showing the relationship between predicted probabilities and observed event rates, calculated using local polynomial regression (LOESS fit). (**C**) Decision curve analysis (DCA) evaluating the net clinical benefit of the model versus default clinical strategies at different threshold probabilities.

A receiver operating characteristic (ROC) curve analysis yielded an area under the curve (AUC) of 0.706 (95% CI, 0.64–0.77; *p* < 0.001), indicating the moderate discriminative capacity of the proposed risk group (RG) model for predicting unfavorable pathology (UP), defined as pathological stage ≥ pT3a, pN+ disease, and/or ISUP grade group ≥ 3 at radical prostatectomy. A calibration analysis demonstrated good agreement between predicted and observed probabilities of UP across the spectrum of predicted risk, with no evidence of substantial systematic over- or underestimation. Decision curve analysis showed that the RG model provided greater net benefit than both the “treat-all” and “treat-none” strategies across a clinically relevant range of threshold probabilities (approximately 30–80%), supporting its potential clinical utility for preoperative risk stratification and surveillance versus surgical decision-making (Figure 2).

### 3.4. Time-to-Recurrence Analysis and Comparison Across Risk Groups

**Figure 3:**
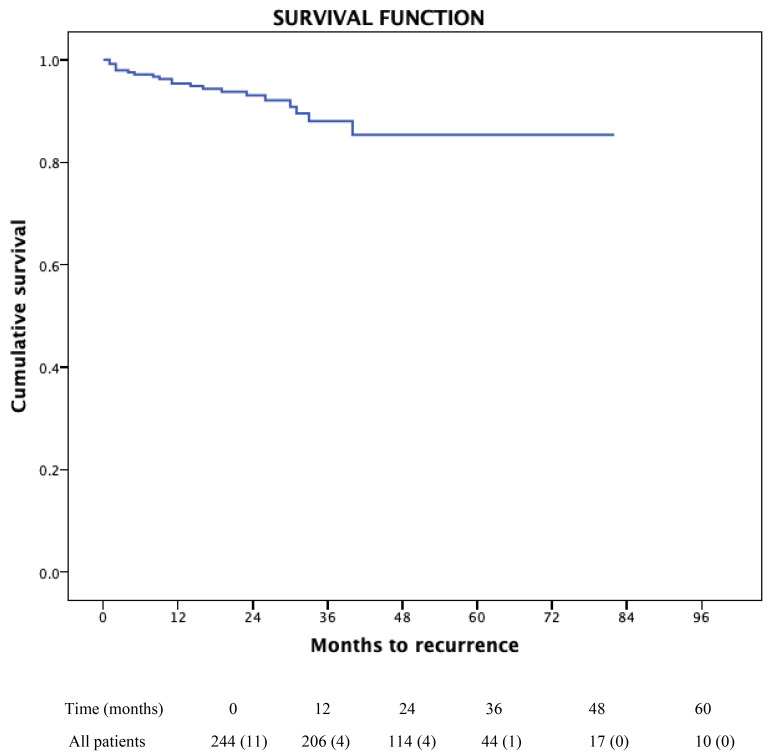
Kaplan–Meier curve depicting recurrence-free survival in the overall cohort. The estimated mean disease-free survival was 73.2 months (95% CI 69.2–77.3). Number of patients at risk (numbers of events).

The median follow-up of the overall cohort was 23 months (range, 1–82 months). During follow-up, 20 patients (8.2%) experienced biochemical recurrence. The median recurrence-free survival was not reached because fewer than 50% of patients developed recurrence during the observation period. The Kaplan–Meier analysis estimated a mean recurrence-free survival of 73.2 months (95% CI, 69.2–77.3) (Figure 3); however, this estimate should be interpreted with caution because of the limited number of recurrence events and the high proportion of censored observations. The estimated recurrence-free survival rates at 12 and 24 months were 95.3% and 93.0%, respectively.

**Figure 4:**
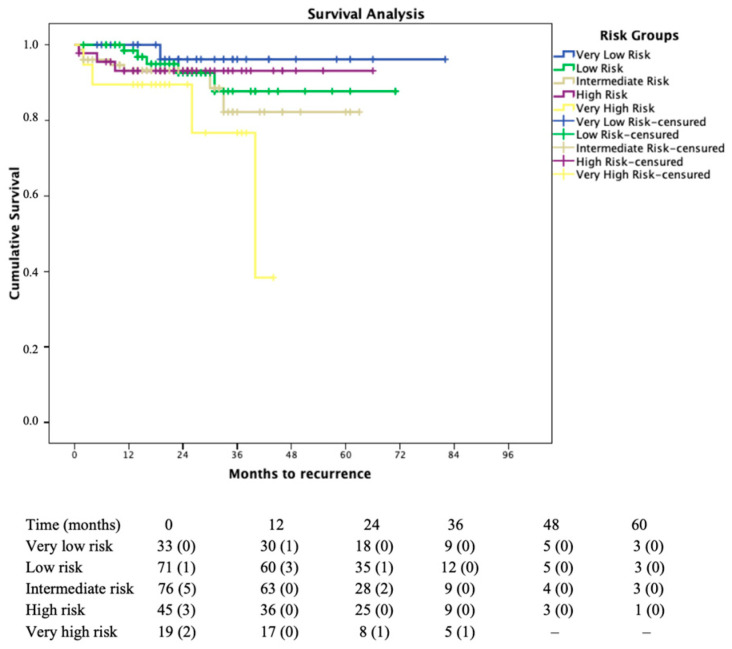
Kaplan–Meier curves stratified by predefined risk clusters with progressively lower recurrence-free survival observed in intermediate- and high-risk groups (*p* = 0.016). Number of patients at risk (number of biochemical recurrence).

Pairwise log-rank tests showed a statistically significant difference in recurrence-free survival only between RG5 and the reference group, RG1 (*p* = 0.016) (Figure 4). No statistically significant differences were observed among the remaining group comparisons. Patients classified within the highest-risk category had shorter recurrence-free survival than those in the very low-risk group.

## 4. Discussion

Large-scale studies [12] have demonstrated that prostate cancer-specific survival does not differ significantly among patients managed with monitoring strategies, surgery, or radiotherapy. However, higher rates of disease progression, treatment escalation, and metastatic events were observed in patients managed with monitoring. Taken together, these findings support the oncologic safety of deferred treatment approaches in appropriately selected patients, while underscoring the importance of accurate risk stratification.

Several consensus statements from the Cancer Guidelines Panel, based on the international DETECTIVE study, indicate that the presence of ISUP grade group 2, PSA > 10 ng/mL, or clinical stage cT2b alone should not automatically exclude patients from AS. These factors should instead be evaluated in conjunction with other clinical parameters before making a final management decision [13].

In this study, we performed an independent multicenter validation of the RG model proposed by Baboudjian et al. [17] to improve risk stratification for patients with IR PCa considered for AS. This classification integrates parameters such as PSAD, PI-RADS score, and DRE findings to complement the European Association of Urology (EAU) criteria [5] and current selection criteria and to estimate the likelihood of occult UP among patients fulfilling expanded AS inclusion criteria. According to Baboudjian et al., the inclusion of patients with PSAD ≥ 0.15 who have a PI-RADS score ≤ 4 (encompassing very low-, low-, and selected intermediate-risk categories within the proposed classification) results in a 253% increase in the number of patients eligible for AS, with only a 27.8% increase in the risk of adverse pathological features [17]; therefore, these observations should be interpreted as estimates of occult unfavorable pathology rather than direct measures of AS outcomes. However, when comparing their results with those of our cohort, notable differences were observed in the proportion of patients with adverse pathological features within each risk subgroup. Specifically, these proportions differed by approximately 10% in the very low- and low-risk categories, with discrepancies reaching up to 15% in the intermediate-risk group. These variations may reflect differences in patient selection or in baseline clinicopathological characteristics and should be considered when interpreting the generalizability of this classification across different clinical settings. Although the present study provides an independent Spanish multicenter validation of the proposed risk classification, some considerations should be acknowledged when interpreting generalizability. The baseline characteristics of our cohort were relatively similar to those of the original derivation cohort, and these findings should be considered to support the reproducibility of the model in an independent multicenter Spanish cohort, rather than as a broad external validation across substantially different geographic, ethnic, or healthcare system contexts. Further studies including more heterogeneous populations are needed before this risk classification can be extrapolated to wider international AS cohorts.

This risk classification may expand the number of patients eligible for AS while integrating readily available parameters routinely used in clinical practice, including mpMRI, PSAD, and DRE findings, to support individualized clinical decision-making. Rather than relying on detailed subclassification of cT2 disease, a simplified distinction based on DRE findings, classifying tumors as clinically non-detectable versus detectable (cT1 versus cT2), may provide greater reproducibility and practicality in routine clinical settings. Magnetic resonance imaging plays an important role in patient selection for AS, as it may serve as an initial tool to identify IR patients suitable for this approach while excluding those with large-volume disease, such as PI-RADS 5 lesions [9], as demonstrated both in the original cohort [17], with an unfavorable pathology detection rate of 54.3% among patients with PI-RADS 5 lesions, and in our cohort, with an unfavorable pathology detection rate of 76.56%, further reinforcing the role of mpMRI and PI-RADS 5 lesions in particular as a robust and reproducible predictor of unfavorable pathology across different clinical settings. Nevertheless, evidence remains limited regarding the incremental contribution of mpMRI, genomic classifiers, or further subclassification of ISUP grade group 2–3 in refining individual risk assessment among IR patients considered for AS [11].

In the near future, genomic markers are likely to be increasingly incorporated into clinical decision-making and, together with established clinical tools, may provide additional support for the personalization of AS strategies. These assays aim to identify early genomic signals associated with tumor progression [18]. The most extensively studied and widely available genomic classifiers include Decipher, Prolaris, the Genomic Prostate Score (GPS), and ProMark, all of which have demonstrated comparable prognostic performance, with reported AUCs ranging from 0.72 to 0.79 [19]. GPS, for instance, has been shown to aid in distinguishing favorable intermediate-risk patients who may benefit from AS versus immediate treatment, as patients with low GPS scores and low tumor volume are more likely to be selected for (and remain on) AS [20]. Similarly, higher Decipher scores have been associated with adverse pathological features in RP specimens and with underlying molecular alterations, including PTEN loss, increased activated CD4 expression, and ERG positivity [21]. Nevertheless, further efforts are required to standardize these assays, prospectively validate their clinical utility, and improve their cost-effectiveness before widespread implementation.

Artificial intelligence (AI) is increasingly supporting the interpretation of biological samples and imaging studies through advanced computational algorithms. Recent evidence supports the integration of routine mpMRI into AS protocols using standardized frameworks, such as the PI-RADS score for lesion characterization and the PRECISE criteria for the assessment of radiologic progression [22]. AI-based machine learning and deep learning models that integrate clinical parameters, including PSA, PSAD, and GGG, with radiologic features have demonstrated high predictive accuracy for histologic or radiologic progression, with reported AUCs exceeding 0.80 [23]. Nevertheless, the clinical applicability of these models in daily practice remains an important consideration. Although the predictive performance of our cluster-based model was lower than that reported for AI-driven approaches (AUC 0.706 vs. >0.80), its design relies on low-cost, readily available variables that are routinely collected in clinical practice, thereby facilitating its implementation across a wide range of healthcare settings. In contrast, AI-based models often require specialized infrastructure, advanced image processing, and substantial technical and economic resources, which may currently limit their widespread clinical adoption. In this regard, despite a comparatively lower predictive accuracy, simpler and more accessible risk stratification tools such as the present model may represent a pragmatic and scalable alternative for patient selection and risk assessment in contemporary active surveillance programs. Complementarily, validated risk calculators such as the Cooperberg Risk Calculator and the Cambridge Model integrate clinical and mpMRI-derived variables to enhance risk stratification, enabling earlier identification of patients at higher risk of progression while potentially reducing unnecessary interventions [24].

Recent studies have highlighted the clinical relevance of pathological upgrading and upstaging in patients with favorable IR prostate cancer. Longoni et al. reported that the majority of patients (76.8%) experienced neither upgrading nor upstaging, whereas 13% showed upgrading, 15% upstaging, and 4.2% both [25]. Importantly, prostate cancer-specific mortality was higher among patients with adverse upgrading or upstaging, particularly in those with the most unfavorable pathological features. These findings suggest that such patients may benefit from closer monitoring or treatment intensification, while individuals without upgrading or upstaging may safely continue standard AS protocols. Consistent with these findings, in our cohort, 180 patients were initially classified as low- or intermediate-risk and subsequently found to have unfavorable pathology (upgrading and/or upstaging), representing 36.7% of the cohort; among these patients, 9.2% developed biochemical recurrence during follow-up.

Traditional progression criteria, such as upgrading from GGG 2 to GGG 3, are limited by their reliance on the relative proportion of Gleason patterns (GP) 3 and 4. To address this limitation, Perera et al. proposed incorporating total GP4 length in addition to the GP 3:4 ratio, recommending treatment initiation when total GP4 increases by more than 1 mm compared with the previous biopsy, provided that the absolute GP4 length is ≥2 mm [26]. This quantitative metric may offer a more nuanced assessment of tumor progression and help refine treatment decisions for IR patients enrolled in AS programs.

While all these news tools are widely available and implemented, we have validated the previously proposed risk stratification for patients with AS expanded criteria showing its putative role in clinical practice. However, certain limitations of this study should be taken into account, as its retrospective design, the relatively small sample size, the absence of centralized mpMRI review and the lack of centralized pathological review, which may have introduced interobserver variability and heterogeneity. Nevertheless, adherence to standardized guidelines and uniform terminology across participating centers likely mitigated potential biases inherent to the multicenter design. In addition, recurrence analyses should be interpreted with caution because only 20 biochemical recurrence events were observed during follow-up and the median follow-up duration was 23 months, limiting the statistical power of time-to-event comparisons.

An additional and important limitation of the present study is related to the scenario of the study population. Our cohort consisted exclusively of patients who underwent RP, rather than patients managed within an AS protocol; consequently, the probability of UP observed in an immediate-surgery cohort cannot be directly equated with the probability of AS failure, metastatic progression, prostate cancer-specific mortality, or long-term loss of tumor control. Therefore, the proposed risk classification should be interpreted as a tool for identifying occult UP at the time of surgery among patients fulfilling expanded AS inclusion criteria, and not as a direct predictor of long-term outcomes during AS. Nevertheless, the use of RP specimens allowed direct assessment of adverse pathological features that may remain underdetected by biopsy, mpMRI, PSA density, and clinical examination at baseline. In this context, the aim of the present study was not to evaluate the oncological safety of AS itself, but rather to assess whether readily available preoperative variables could help identify patients with apparently favorable or intermediate-risk disease who harbor occult UP.

Another limitation is the incomplete data availability regarding the total number and detection rate of lymphadenectomies. Although unfavorable pathology was defined as ≥pT3a and/or pN+ and/or ISUP grade group ≥3, the number of patients undergoing pelvic lymph node dissection was not uniformly collected across centers, although the centers had to specify whether the patients were pN+. Therefore, unfavorable pathology in this cohort should be interpreted mainly as reflecting adverse local pathological features.

## 5. Conclusions

The proposed RG classification demonstrated moderate predictive ability for identifying occult unfavorable pathology in radical prostatectomy specimens among surgically treated patients fulfilling expanded AS inclusion criteria. These findings suggest that the model may contribute to baseline risk assessment when counseling patients about management options. However, because the model was developed and validated exclusively in prostatectomy cohorts, its ability to predict active surveillance progression, treatment conversion, or long-term oncological outcomes remains unknown and requires prospective validation in cohorts managed with active surveillance.

## Figures and Tables

**Table 1:** Baseline characteristics of the study cohort (*n* = 244).

	Global Cohort (*n* = 244)	Favorable Features at Final Pathology (*n* = 129)	Adverse Features at Final Pathology (*n* = 115)	*p*
Age; years	63 (57.25–66.00)	62 (57.25–65.00)	63 (57.25–66.75)	0.201
PSA, ng/mL				0.006
<10	204 (83.6%)	117 (90.7%)	87 (75.7%)	
10–15	31 (12.7%)	9 (7%)	22 (19.1%)	
>15	9 (3.7%)	3 (2.3%)	6 (5.2%)	
PSA density, ng/mL^2^				0.214
<0.15	136 (55.7%)	77 (59.7%)	59 (51.8%)	
≥0.15	108 (44.3%)	52 (40.3%)	56 (48.7%)	
T stage				0.000
cT1	204 (83.6%)	119 (92.2%)	85 (73.9%)	
cT2	40 (16.4%)	10 (7.8%)	30 (26.1%)	
mpMRI Results				0.000
PI-RADS < 3	32 (13.1%)	23 (17.8%)	9 (7.9%)	
PI-RADS 3	24 (9.8%)	17 (13.2%)	7 (6.1%)	
PI-RADS 4	124 (50.8%)	74 (57.4%)	50 (43.5%)	
PI-RADS 5	64 (26.2%)	15 (11.6%)	49 (42.6%)	
ISUP group				0.000
ISUP 1	98 (40.2%)	67 (51.9%)	31 (27%)	
ISUP 2	146 (59.8%)	62 (48.1%)	84 (73%)	
Unfavorable pathology (UP)	115 (47.1%)			
Risk Group Classification (RG)				0.000
Very low risk (RG1)	33 (13.5%)	28 (84.8%)	5 (15.2%)	
Low risk (RG2)	71 (29.1%)	42 (59.2%)	29 (40.8%)	
Intermediate risk (RG3)	76 (31.1%)	44 (57.9%)	32 (42.1%)	
High risk (RG4)	45 (18.4%)	13 (28.9%)	32 (71.1%)	
Very high risk (RG5)	19 (7.8%)	2 (10.5%)	17 (89.5%)	

Data are presented as median (interquartile range) or number (percentage). Abbreviations: PSA—prostate-specific antigen; cT—clinical tumor stage; mpMRI—multiparametric magnetic resonance imaging; PI-RADS—Prostate Imaging Reporting and Data System; ISUP—International Society of Urological Pathology; UP—unfavorable pathology; RG—risk group.

**Table 2:** Risk of unfavorable pathology across risk groups: results of ordinal and categorical regression analyses.

Ordinal Analysis		
Risk Group	Global OR (95%CI)	Increase in the Risk of Unfavorable Pathology Compared with RG1 *
RG1	2.141 (95%CI 1.639–2.795; *p* < 0.001)	Reference
RG2		2.141-fold higher risk
RG3		4.584-fold higher risk
RG4		9.814-fold higher risk
RG5		21.012-fold higher risk
Categorial analysis		
Risk group	OR (95%CI)	*p*-value
RG1	1.000 (Reference)	—
RG2	3.867 (1.336–11.191)	0.013
RG3	4.073 (1.418–11.696)	0.009
RG4	13.785 (4.368–43.507)	<0.001
RG5	47.600 (8.296–273.112)	<0.001

* Increase in risk estimated while holding all other variables constant and assuming no interaction. Abbreviations: RG—risk group; CI—confidence interval.

## Data Availability

The data presented in this study are available from the corresponding author upon reasonable request. The data are not publicly available due to patient confidentiality and privacy restrictions.

## Supplementary Materials

The following supporting information can be downloaded at: https://www.mdpi.com/article/10.3390/diagnostics16132073/s1. Table S1. Baseline characteristics of the study cohort stratified by participating center.
