# Supplementary material for: Multicenter Validation of a Risk Classification Cluster for Unfavorable Pathology in Prostatectomy Specimens of Patients with an Active Surveillance Expanded Inclusion Criteria
Source: Diagnostics (Basel). 2026 Jul 2;16(13):2073. doi: 10.3390/diagnostics16132073 (PMC13360441; doi:10.3390/diagnostics16132073)
Supplement: Supplementary file 1 [file diagnostics-16-02073-s001.zip › diagnostics-4379703-supplementary.pdf]

SUPPLEMENTARY TABLE

Supplementary Table S1. Baseline characteristics of the study cohort stratified by participating center.

Variable	Córdoba (n=164)	Málaga (n=36)	Sevilla (n=44)
**PSA, ng/mL**			
**<10**	139 (84.8%)	31 (86.1%)	34 (77.3%)
**10–15**	21 (12.8%)	3 (8.3%)	8 (18.2%)
**>15**	4 (2.4%)	2 (5.6%)	2 (4.5%)
**PSA Density, ng/mL²**			
**<0.15**	104 (63.4%)	16 (44.4%)	13 (29.5%)
**≥0.15**	60 (36.6%)	20 (55.6%)	31 (70.5%)
**T Stage**			
**cT1**	133 (81.1%)	34 (94.4%)	37 (84.1%)
**cT2**	31 (18.9%)	2 (5.6%)	7 (15.9%)
**ISUP Grade**			
**ISUP 1**	62 (37.8%)	4 (11.1%)	32 (72.7%)
**ISUP 2**	102 (62.2%)	32 (88.9%)	12 (27.3%)
**PI-RADS Score**			
**<3**	21 (12.8%)	1 (2.8%)	10 (22.7%)
**3**	16 (9.8%)	5 (13.9%)	3 (6.8%)
**4**	77 (47.0%)	28 (77.8%)	19 (43.2%)
**5**	50 (30.5%)	2 (5.6%)	12 (27.3%)
**Unfavorable Pathology**			
**No**	64 (39.0%)	28 (77.8%)	37 (84.1%)
**Yes**	100 (61.0%)	8 (22.2%)	7 (15.9%)
